# “Case series: ischemic stroke associated with dehydration and arteriosclerosis in individuals with severe anorexia nervosa”

**DOI:** 10.1186/s40337-021-00393-w

**Published:** 2021-03-20

**Authors:** Yu Mimura, Yusuke Shimizu, Hiroki Oi, Shin Kurose, Shun Kudo, Taketo Takata, Masaru Mimura, Michitaka Funayama

**Affiliations:** 1 Department of Neuropsychiatry, Japanese Red Cross Ashikaga Hospital, 49-1 Yobe-Cho, Ashikaga-shi, Tochigi, 326-0843 Japan; 2 Department of Neuropsychiatry, Keio University School of Medicine, 35 Shinanomachi, Shinjuku-ku, Tokyo, 160-8582 Japan

**Keywords:** Anorexia nervosa, Ischemic stroke, Dehydration, Hypoperfusion, Arteriosclerosis, Hypercoagulability, Case report

## Abstract

**Background:**

Numerous reports have indicated that patients with anorexia nervosa (AN) are at a relatively high risk of developing vascular diseases, including cardiovascular events and venous thromboembolism. However, there have been no previous reports of the development of ischemic stroke during refeeding therapy in patients with severe AN. This report is aimed at reporting the characteristics of an ischemic stroke in patients with AN.

**Case presentations:**

Our study included 29 admissions by independent 19 female patients cases (19 patients), who received thorough medical, neurological, and psychiatric examinations. Two patients were diagnosed as having developed ischemic stroke; the first patient showed multiple infarctions in the brain, while the second showed symptomatic focal infarction. Our findings suggest that dehydration and arteriosclerosis, in association with severe malnutrition, could predispose to the development of ischemic stroke in patients with severe AN.

**Conclusions:**

Development of ischemic stroke in patients with AN might be overlooked. Watching out for neurological signs would help in early diagnosis of ischemic stroke in patients with AN during refeeding. Specific etiology could induce ischemic stroke in patients with AN even if they have no common risk factors of ischemia.

## Background

Anorexia nervosa (AN) is a life-threatening psychosomatic condition, in which patients frequently develop severe complications, including hepatic dysfunction [1], gastrointestinal problems [2], respiratory failure [3], and cardiac disease [4]. Some complications eventually lead to fatal outcomes despite careful treatments [5].

As for the vascular complications in patients with AN, these patients are at an elevated risk of developing cardiac events due to early arteriosclerotic damage [6]. In addition, recent case series have suggested that the development of venous thromboembolism is often overlooked in patients with severe AN [7]. Taken together, these previous reports suggest that patients with severe AN frequently have arteriosclerotic damage and venous stasis. Therefore, it is important to suspect vascular diseases, both arterial and venous, so that these complications would be diagnosed early and treated promptly.

In general, the causes of ischemic stroke are classified under four categories: atherosclerosis, cardiac embolism, small vessel disease, and others [8]. Considering the high risk of vascular diseases in patients with AN, we can assume that patients with severe AN would also have a high risk of developing ischemic stroke. However, to the best of our knowledge, there have been no published reports of patients with AN presenting with an ischemic stroke.

For this study, all admitted 29 admissions cases with severe AN who needed emergent nutritional therapy and were admitted to our hospital between April 2018 and November 2020 were reviewed, and two of these patients developed ischemic stroke during their hospital stay. Among these 29 admissions cases by 19 independent female patients (two patients were admitted four times, and four patients were admitted two times.), they were all diagnosed with AN according to the diagnostic and statistical manuals of mental disorders fifth edition (DSM-V), and 16 patients were AN-restricted type, while 3 patients were AN-binge-eating/ purging type. Age and BMI at admission (Mean ± SD) were 33.6 ± 11.6 and 11.4 ± 1.56, respectively.

This first report of two cases aims to draw attention to the high risk of development of ischemic stroke in patients with severe AN, because our cases showed only subtle neurological deficits. Clinicians need to watch carefully for the development of any neurological symptoms, including hemiplegia and linguistic impairment, in patients with severe AN receiving refeeding therapy, because these symptoms can sometimes be overlooked in clinical settings.

## Case presentation

### Patient 1

**Fig. 1:**
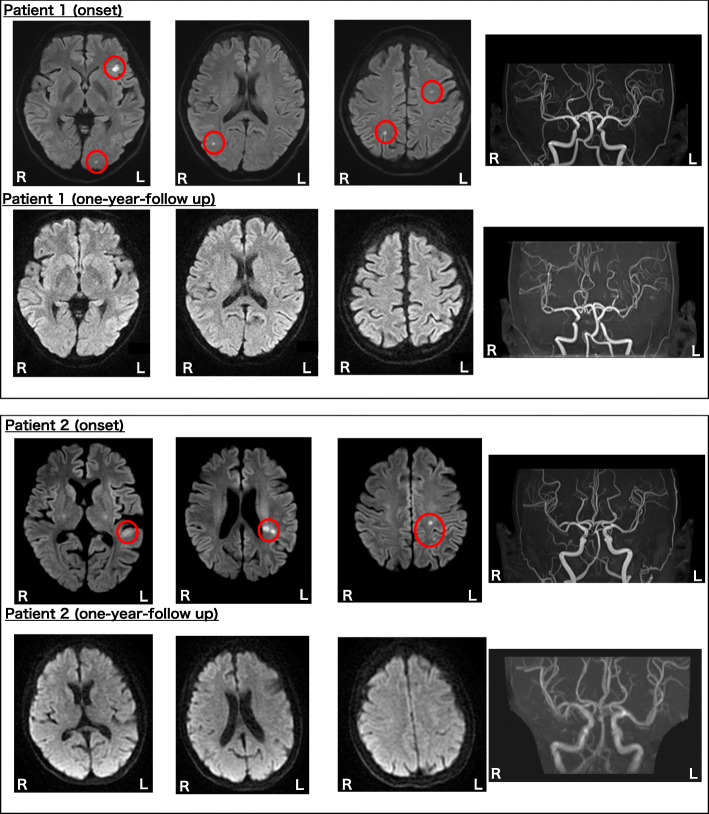
Brain MRI (Patient 1, 2). Patient 1 (Upper row) Brain diffusion-weighted MRI revealed disseminated cortical infarctions bilaterally. MR angiography showed no arterial stenosis. (Lower low) Brain diffusion-weighted MRI and MR angiography (one year after the discharge) revealed no findings. Patient 2 (Upper row) Brain diffusion-weighted MRI revealed focal brain infarction in the white matter underlying the left temporal transverse temporal gyrus, the left supramarginal gyrus, and the left parietal cortex. MR angiography showed no arterial stenosis. (Lower low) Brain diffusion-weighted MRI and MR angiography (one year after the discharge) revealed no findings

Patient was a 31-year-old woman with a 10-year history of the binge eating/purging type of AN. S She had no past history of neurological or cardiovascular diseases, and well-known risk factors such as dyslipidemia, hypertension, and diabetes mellitus. Also, she had no family history of stroke. She was a current smoker, with a smoking history of one pack-year. She was admitted to our hospital with a history of easy fatigability and immobility. She was unable to walk without assistance; however, her consciousness was clear, and full neural examinations revealed no signs of neurological deficits. We diagnosed her as having severe malnutrition, as her body mass index (BMI) was 8.8 (height: 1.64 m, weight: 23.4 kg). Refeeding therapy was initiated at 2000 kcal/day with adequate electrolyte supplementation, during which she showed no complications; the electrolyte levels remained under good control, along with normal blood glucose levels. Water intake was restricted to 1 ml/1 kcal, and there were no complications such as congestive heart failure. On the 10th hospital day, she complained of dysesthesia on her left shoulder. The patient was alert, attentive, and oriented. The cranial nerve was intact. The Barre and Mingazzini test revealed that both flexors and extensors of the left upper and lower extremities were weaker than the right upper and lower ones. The results of manual muscle testing were as follows (R/L); deltoid 5/4+, biceps 5/4+, triceps 5/4+, wrist flexor 5/4, wrist extensor 5/4, hip flexor 5/4+, hip extensor 5/4+, knee flexor 5/4+, knee extensor 5/4+, ankle flexor 5/4+, ankle extensor 5/4+. The tendon reflexes of the extremities were symmetrical, and no abnormal reflexes were found. There was no dysmetria on a finger-to-nose test. Sensory examination reveals hypesthesia to touch and pain on her left shoulder to fingers. But her neurological symptoms resolved spontaneously within 10 min. Her vital signs were as follows: HR 74/min, BP 86/53 mmHg, BT 36.7 °C. Brain magnetic resonance imaging (MRI) revealed disseminated infarctions in the cortex bilaterally; however, her motor symptoms were not explained by the imaging findings (Fig. 1). We diagnosed her as having had a transient ischemic attack, with incidental detection of multiple infarctions. Extensive clinical workup, including blood test (biochemistry, blood count, coagulation profile, and autoimmune profile.), ultrasound (carotid artery and lower extremities vein), whole-body computation tomography, Holter electrocardiogram, echocardiography, ankle-brachial index (ABI), and cardio-ankle vascular index (CAVI), to determine the etiology of ischemia revealed that she had no systemic atherosclerosis or any source of embolism. However, her ABI and CAVI values were elevated (Table 1), suggesting that she had arteriosclerotic damage without atherosclerosis. Laboratory tests performed on the day that she was diagnosed as having a stroke excluded common causes of early-onset ischemic stroke (vasculitis, dissection, moyamoya disease, antiphospholipid antibody syndrome). Elevated BUN/Cre levels suggested that she had dehydration, and the elevated serum levels of thrombin-antithrombin complex (TAT-III), platelet factor 4 (PF4), and β-thromboglobulin (βTG) indicated that she was in a hypercoagulable state (Table 1). She was started on aspirin 100 mg per day for secondary prevention. After the refeeding therapy, she was discharged, and developed no recurrence of the ischemic attack during the subsequent two-year follow-up period. We checked up her head MRI scan one year after her discharge with no findings.

**Table 1:** Laboratory data (Patient 1, 2)

		Pt 1				Pt 2				Reference value
		Admission	Onset	Nadir During First two weeks	Discharge	Admission^a^	Onset	Nadir During First two weeks	Discharge	
Biochemistry	BUN (mg/dL)	40.9	13.3	12.2	19.8	23.3	23.3	17.7	9.6	8–20
	Cre (mg/dL)	0.73	0.32	0.30	0.42	0.55	0.55	0.55	1.29	0.46–0.79
	BUN/Cre	56.03	41.56	NA	47.14	42.36	42.36	NA	7.44	
	Glucose (mg/dL)	177	86	80	103	99	99	89	90	73–109
	Na (mmol/L)	135	142	135	139	137	137	137	141	138–145
	Cl (mmol/L)	88	112	88	109	96	96	96	105	101–108
	K (mmol/L)	2.8	4.5	2.8	4.3	3.4	3.4	3.4	3.4	3.6–4.8
	Ca (mg/dL)	7.7	7.7	7.7	8.8	8.0	8.0	7.9	8.3	8.8–10.2
	Mg (mg/dL)	2.8	1.5	1.4	1.8	1.8	1.8	1.8	3.0	1.8–2.4
	P (mg/dL)	3.6	2.4	2.2	4	4.5	4.5	4.5	5.1	2.4–4.6
	AST	244	52	26	23	46	46	37	15	
	ALT	135	87	51	40	88	88	19	12	
	CRP	< 0.01	< 0.01	< 0.01	< 0.01	< 0.10	< 0.10	< 0.10	< 0.10	
	TC (mg/dL)	195	155	155	191	212	212	212	295	142–248
	TG (mg/dL)	24	58	24	47	71	71	71	151	30–117
	HDL-C (mg/dL)	119	65	65	89	82	82	82	90	48–103
	LDL-C (mg/dL)	77	81	77	91	122	122	122	181	65–163
	FT3 (pg/mL)	< 1.5	2.24	< 1.5	NA	2.05	2.05	2.05	1.79	1.88–3.18
	FT4 (ng/dL)	1.31	0.74	0.74	NA	0.72	0.72	0.72	0.82	0.7–1.4
	TSH (μIU/mL)	2.04	1.18	1.18	NA	1.24	1.24	1.24	0.28	0.35–4.94
	Hb-A1c (%)	5.8	5.8	5.8	NA	6.2	6.2	6.2	4.9	4.9–6.0
	BNP (pg/mL)	41.9	50.4	41.9	NA	NA	NA	NA	NA	0–18.41
Blood count	WBC (/μL)	4200	2500	2300	4600	10,500	10,500	4200	8700	3300–8600
	RBC (X10^4/μL)	392	260	233	333	286	286	262	372	386–492
	Hb (g/dL)	13.3	9.1	8.1	11.8	7.5	7.5	6.9	7.8	11.6–14.8
	Hct (%)	38.3	27.5	25.4	37.4	24.3	24.3	23.0	27.5	35.1–44.4
	Plt (X10^4/μL)	15.5	14.6	9.5	21.3	54.3	54.3	49.1	54.5	
Coagulation profile	PT-INR	1.49	1.14	1.14	NA	0.99	0.99	0.93	NA	0.8–1.2
	APTT (sec)	29.3	24.6	24.6	NA	23.3	23.3	18	37.7	23.5–31.5
	D-dimer (μg/mL)	< 0.25	1.2	< 0.25	NA	1.7	1.7	1.2	0.6	0–1
	TAT III (ng/L)	NA	26.9	26.9	3.6	3.6	3.6	3.6	NA	0–3
	Protein C activity (%)	NA	57	57	NA	97	97	97	NA	64–146
	Protein S (%)	NA	84	84	NA	120	120	120	NA	60–150
	PF4 (ng/mL)	NA	33	33	70	24	24	24	NA	0–20
	βTG (ng/mL)	NA	104	104	129	105	105	105	NA	0–50
Autoimmune antibodies^b^	ANA	20				< 20				< 20
	PR3-ANCA (U/mL)	< 1.0				< 1.0				0–3.5
	MPO-ANCA (U/mL)	< 1.0				< 1.0				0–3.5
	Anti-cardiolipin antibody (U/mL)	< 10				8				0–10
Imaging findings^b^	Ultrasound (carotid artery)	no atherosclerosis				no atherosclerosis				
	Ultrasound (lower extremity veins)	no embolism				no embolism				
	Whole-body CT	no evidence of malignancy				no evidence of malignancy				
	Holter ECG	no evidence of Af				no evidence of Af				
	Echocardiography	EF 70% Wall motion normal No embolism				EF 70% Wall motion normal No embolism				
CAVI/ABI^b^	CAVI (R/L)	7.8/7.7				7.1/6.9				
	ABI (R/L)	0.77/0.83				1.04/1.05				
CSF^b^	CSF cell count (/μL)	NA				1				0–5
	CSF protein (mg/dL)	NA				24				15–45
	CSF IgG-index	NA				0.19				0–0.7
	CSF MBP	NA				negative				negative
	CSF OCB	NA				negative				negative

^a^ In patient2, the values on ischemic stroke onset are same as on admission

^b^ For autoimmune antibodies, imaging findings, CAVI/ABI, and CSF, only the values on ischemic stroke onset are shown in the table

### Patient 2

**Fig. 2:**
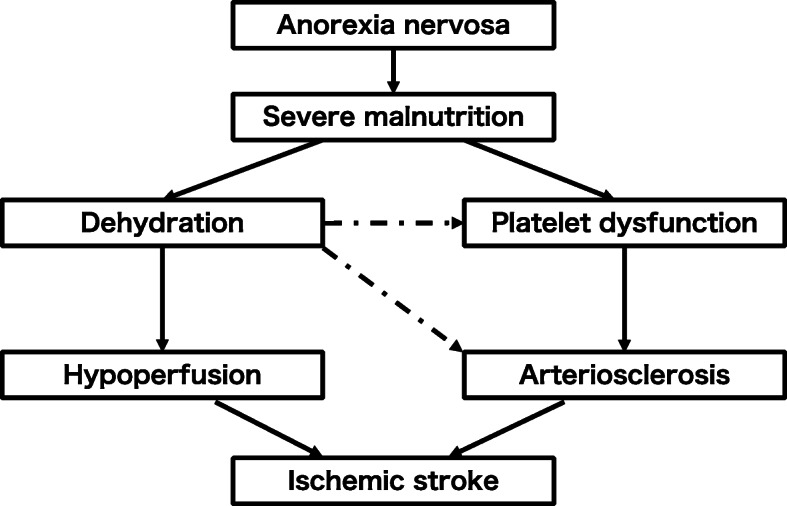
Suggested relationships among hypoperfusion, arteriosclerosis, and malnutrition due to anorexia nervosa. The suggested relationships among dehydration, arteriosclerosis, and severe malnutrition are quite complex. Severe malnutrition in association with severe AN may induce hypoperfusion due to dehydration and arteriosclerosis in association with platelet dysfunction. Moreover, hypovolemia caused by dehydration can induce arteriosclerosis and dehydration itself may be associated with impaired endothelial function

Patient was a 50-year-old woman with a 13-year history of the binge eating/purging type of AN. She had no past history of neurological or cardiovascular diseases, smoking, and well-known risk factors such as dyslipidemia, hypertension, and diabetes mellitus. She had no family history of stroke. She was diagnosed as having severe malnutrition and hospitalized, as her BMI was 10.4 (height; 1.57 m, weight; 25.6 kg). Her vital signs were as follows: HR 103/min, BP 88/65 mmHg, BT 37.0 °C. On admission, she had difficulty in speaking and word comprehension, as a result of phonological errors and word-sound deafness, respectively. Her speech production concerning articulation was fluent. She, however, sometimes had word-finding difficulties along with phonological errors. Due to the phonological dysfunction, she sometimes stuttered and repeated the initial letter of a word. Although she had problems with speech, she was alert, attentive, and oriented. The cranial nerve was intact. She showed no paralysis. The results of manual muscle testing were all 5/5. The tendon reflexes of the extremities were symmetrical, and no abnormal reflexes were found. There was no dysmetria on the finger-to-nose test. She complained of no sensory disturbance. Brain MRI revealed a high-intensity area in the white matter of the left temporal-parietal lobe, underlying the left supramarginal gyrus as well as the left transverse temporal gyrus (Fig. 2), which explained her phonological errors and word-sound deafness, respectively. We diagnosed her as having symptomatic cerebral infarction. Extensive clinical workup and laboratory tests performed to determine the etiology of the ischemia on the day that she was diagnosed as having a stroke revealed that she had no systemic atherosclerosis, no source of embolism, or none of the common causes of early-onset ischemic stroke, just like in patient 1. Also, like patient 1, she showed elevated levels of TAT-III, βTG, and PF4, suggesting that she was in a hypercoagulable state (Table 1). Unlike patient 1, however, she showed no elevation of the CAVI or ABI value. On the other, she also showed elevated BUN/Cre levels, suggestive of dehydration. The patient was therefore initiated on aspirin 100 mg daily for secondary prevention. Refeeding therapy at 1800 kcal/day and water restriction to 1 ml/1 kcal was successful, and after an uneventful course without the development of any complications, the patient was discharged while showing gradual improvement of her linguistic functions. We checked up her head MRI scan one year after her discharge with no findings, however, she had a generalized convulsion due to the ischemic stroke, and the convulsions were well controlled with levetiracetam at the dose of 1000 mg per day during the subsequent follow-up period of a year. Her mild word-finding difficulties and word-sound deafness had almost disappeared.

## Discussion and conclusions

From this case series, we report two patients with severe AN who presented with ischemic stroke associated with a hypercoagulable state. These cases provide novel insights: clinicians should suspect development of ischemic stroke in patients with severe AN receiving care for severe malnutrition, and specific approaches such as rehydration would be required in AN patients with an ischemic stroke.

First of all, our case reports suggest that patients with severe AN are at a higher risk of developing ischemic stroke than the general population. Based on the estimated annual incidence of juvenile (age 15–49 years) stroke of 10.8/100000 (range 8.4 to 13.0) and men being at a higher risk than women [9], the incidence rate in our patients (2/19) seemed to be higher than that in the general population. Our cases suggest that careful management, especially in the presence of neurological deficits, is needed for patients with severe AN.

In general, dehydration is thought to be involved in the occurrence of ischemia. Some researchers have suggested that dehydration may be an independent risk factor for in-hospital/postoperative ischemic stroke [10] [11]. Moreover, dehydration combined with low blood pressures seems to induce cerebral hypoperfusion, which can exacerbate ischemic stroke [12]. Our cases presented here showed high values of the BUN/Cre ratio (approximately 40) associated with a low blood pressure, suggesting that dehydration was the primary pathophysiologic mechanism underlying the development of ischemic stroke. However, there are currently no consensus diagnostic criteria for dehydration in patients with stroke. The BUN/Cre ratio is the most commonly used laboratory marker of dehydration [12]; meanwhile, the BUN/Cre may overestimate dehydration in patients with AN, due to the decreased muscle volume [13]. The values of BUN were nearly within normal range in our cases, indicating that other possible mechanisms might also exist. According to previous reports, dehydration was associated with a poor prognosis and functional outcome after acute ischemic stroke [14], and early rehydration therapy during acute ischemic stroke could improve the prognosis and the functional outcome [15]. However, we were unable to apply these findings to patients with AN, since congestive heart failure is a common complication associated with refeeding syndrome [4]. In our cases, water restriction to 1 ml/1 kcal was well tolerated, without the development of any cardiac complications. Rehydration therapy with careful monitoring is necessary, and further studies are warranted to establish the prevalence of dehydration and design a rehydration protocol for acute ischemic stroke in patients with severe AN.

In our presented cases, the elevated levels of βTG4/PF4 and TAT-III reflected increased platelet activation and thrombin formation, respectively, indicating that lacunar infarction was unlikely in our patients, since lacunar infarction is not known to be associated with a hypercoagulable state [16–18]. Our extensive workup to determine the etiology of the cerebral infarction revealed no source of embolism. Based on these profiles, this case report suggests that ischemic stroke in cases of severe AN seems to be caused by arteriosclerosis. In fact, patient 1 might have had arteriosclerosis, because her CAVI and ABI were elevated. A previous report indicated that patients with AN had a decreased platelet count, suggestive of dysregulated thrombopoiesis [19], which may induce arteriosclerosis. Moreover, the U-shaped relationships between platelet counts and risk of ischemic stroke have been reported [20], suggesting that patients with increased platelet count such as patient 2 could have higher risk of ischemic stroke. As for dysfunction of the coagulation system, little has been reported on parameters of the coagulation profile, especially the plasma levels of thrombin, in patients with AN. Our cases presented here suggest that severe AN patients have systemic arteriosclerosis due to impaired platelet function and coagulopathy, which is consistent with previous reports [6,7].

The relationships among dehydration, arteriosclerosis, and severe malnutrition are quite complex. As described above, undernourishment in association with severe AN may induce hypoperfusion due to dehydration and arteriosclerosis in association with platelet dysfunction. On the other hand, hypovolemia caused by dehydration can elevate the plasma aldosterone level, which has been linked to vascular stiffening [21], and dehydration itself may be associated with cardiovascular disease through impaired endothelial function [22]. These ideas are summarized in Fig. 2.

This case report includes several limitations. First of all, this study was conducted at a single general hospital. Multicenter studies are warranted to establish the best treatments for ischemic stroke in patients with AN. Second, not all patients included in our patients had undergone MRI assessments, because of insufficient equipment. Third, ischemic stroke secondary to paradoxical embolism could not be excluded, because transesophageal echocardiography was not performed in the patients; however, we consider it as having been unlikely as our patients showed relatively low serum D-dimer levels.

In conclusion, we report here two patients with severe AN with ischemic stroke caused by hypoperfusion and partial arteriosclerosis associated with severe malnutrition. Our extensive clinical workup to determine the etiology of ischemia just revealed a hypercoagulable state, without any apparent embolic or atheromatous source. Further extensive group studies or group-based studies are needed to elucidate the etiology of ischemic stroke in patients with severe AN.

## Data Availability

All data generated or analyzed during this study are included in this published article and its. supplementary information files.

## Abbreviations

AN: Anorexia nervosa
BMI: Body mass index
TAT-III: Thrombin-antithrombin complex
PF4: Platelet factor 4
βTG: β-thromboglobulin
ABI: Ankle-brachial index (ABI)
CAVI: Cardio-ankle vascular index (CAVI)
BUN: Blood urea nitrogen
Cre: Creatinine
TC: Total cholesterol
TG: Triglyceride
HDL-C: High density lipoprotein cholesterol
LDL-C: Low density lipoprotein cholesterol
BNP: Brain natriuretic peptide
PT-INR: Prothrombin time international normalized ratio
APTT: Activated partial thromboplastin time
ANA: Anti-nuclear antibody
PR3: Proteinase 3
MPO: Myeloperoxidase
ANCA: Anti-neutrophil cytoplasmic antibody
ECG: Electrocardiogram
EF: Ejection fraction
Af: Arterial fibrillation
CSF: Cerebrospinal fluid
MBP: Myelin basic protein
OCB: Oligoclonal band
